# Quality assessment of microarrays: Visualization of spatial artifacts and quantitation of regional biases

**DOI:** 10.1186/1471-2105-6-166

**Published:** 2005-07-01

**Authors:** Mark Reimers, John N Weinstein

**Affiliations:** 1 Genomics & Bioinformatics Group, Laboratory of Molecular Pharmacology, Center for Cancer Research, National Cancer Institute, Bethesda Maryland, 20892 USA

## Abstract

**Background:**

Quality-control is an important issue in the analysis of gene expression microarrays. One type of problem is regional bias, in which one region of a chip shows artifactually high or low intensities (or ratios in a two-channel array) relative to the majority of the chip. Current practice in quality assessment for microarrays does not address regional biases.

**Results:**

We present methods implemented in R for visualizing regional biases and other spatial artifacts on spotted microarrays and Affymetrix chips. We also propose a statistical index to quantify regional bias and investigate its typical distribution on spotted and Affymetrix arrays.

We demonstrate that notable regional biases occur on both Affymetrix and spotted arrays and that they can make a significant difference in the case of spotted microarray results. Although strong biases are also seen at the level of individual probes on Affymetrix chips, the gene expression measures are less affected, especially when the RMA method is used to summarize intensities for the probe sets. A web application program for visualization and quantitation of regional bias is provided at .

**Conclusion:**

Researchers should visualize and measure the regional biases and should estimate their impact on gene expression measurements obtained. Here, we (i) introduce pictorial visualizations of the spatial biases; (ii) present for Affymetrix chips a useful resolution of the biases into two components, one related to background, the other to intensity scale factor; (iii) introduce a single parameter to reflect the global bias present across an array. We also examine the pattern distribution of such biases and conclude that algorithms based on smoothing are unlikely to compensate adequately for them.

## Background

Microarrays and other new high-throughput technologies are changing the way molecular biology is practiced. However microarray platforms and protocols are still under development, and the causes of common errors and artifacts are still not completely understood or controlled. Over time and replications, many types of errors seem almost random. Others, however, affect many gene expression measures at once, introducing systematic biases into the data. Most statistical methods are designed to deal with measures corrupted by random noise; methods to deal with systematic biases are not so well developed.

### Spotted arrays

Over the extent of a single spotted microarray, factors such as temperature, liquid flow rate or RNA diffusion rate may differ among different regions on the array. Often, washing is less thorough near the edges of a slide, contributing to higher local off-spot background near the edges. High local background usually pushes up the spot measures, although not always predictably. To detect such technical artifacts, it is now standard practice to examine images of slides for pronounced irregularities and high backgrounds. Such examination can identify many types of faults, but even a skilled technician may miss regions of higher than average intensity in Affymetrix arrays or moderate biases in the ratios calculated for spotted arrays, since the vast majority of spots appear dim in images calibrated for the dynamic range of the brightest spots. At the moment there is no way of quantifying regional biases, and a lot is left to the technician's judgment.

### Affymetrix arrays

To address such issues, Affymetrix has gone to great lengths to standardize their procedure. However, uniform results are rarely achieved in practice. Often a bubble remains after filling an Affymetrix cassette. This bubble will not travel uniformly over the chip during hybridization mixing and may get stuck or move in an irregular circuit. Scratches and other manufacturing imperfections can make a difference. Scratches are sometimes visible with the aid of software such as dChip [1] or RMA [2]. Although a skilled technician can identify some of the grosser faults by examining the images of hybridized Affymetrix chips, he or she has no current standard for measuring how serious the problems are or for knowing whether other sorts of systematic problems are evading scrutiny.

Current practice in quality assessment for spotted arrays considers individual spot measures, such as area and signal/noise ratio [3]. Current quality metrics for Affymetrix arrays consider 3'/5' ratios for selected genes and spike-in ratios. These quality metrics don't take variation within a slide or chip into account. We show here that such bias, which is currently ignored, can be a significant problem.

## Methods

All computation was done within the R programming environment [11], and the Affymetrix analysis used the affy package [12].

### Detection of regional bias on spotted arrays

**Figure 1:**
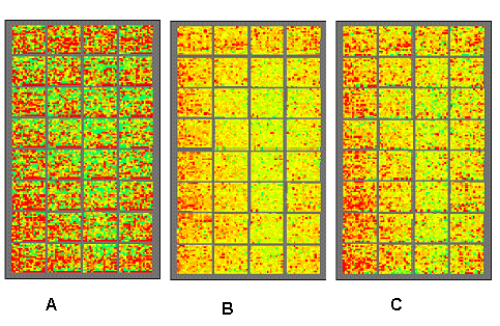
**The advantages of using a reference to highlight regional biases. **Figure 1A shows log_2 _spot ratios at a constant intensity. Red corresponds to a log ratio of greater than 0.5; yellow to a log ratio of 0, and green to a log ratio of less than -0.5. Figure 1B shows the log_2 _ratios for the same slide relative to the averages of the log_2 _ratios across all the slides. Figure 1C shows the log ratios for the same slide after background subtraction. The right portion of the bottom row in each print-tip group was spotted with buffer only.

An effective way to present information about regional biases is through plots or maps of the ratios or signals over the chip surface. For two-color arrays it is natural to plot ratios as a function of position. Because all ratios are represented at the same brightness, such a plot makes it much easier to see patterns of regional bias than does inspection of the raw image file. Such a plot is shown in Figure 1A. Similar plots are available through arrayMagic [4]. However we still face the problem that many different ratios (high and low) are juxtaposed on the slide, making it difficult to see subtle but consistent biases.

Many two-color microarray experiments focus on a single tissue but use a common reference RNA not specific to the tissue. In such a design, two neighboring probes will often show consistently different red/green ratios across all slides, reflecting the typical abundance of the probes' mRNA targets in the samples relative to the common reference. We would like to compare each slide's probe ratios with a standard ratio profile, reflecting the typical abundance of all mRNA species in the tissue under study relative to the reference. We approximate such a common standard by computing, for each probe, the 20%-trimmed mean of the probe's log ratios, across all slides. In doing so, we are assuming that the biological variation due to sample and the regional biases on each slide will tend to balance out over the whole experimental set.

For each slide we then compute the difference between the log ratio of each spot and the spot's average log ratio over all of the pertinent arrays:

(1) d_i,j _= log_2_(R_i,j_/G_i,j_) – m_i_; m_i _= trim(log_2_(R_i,k_/G_i,k_)),

where d is the difference, i indexes the spot, j indexes the particular slide, k indexes all slides, and trim refers to the 20% trimmed mean of a set; Ri,j and Gi,j are the red and green channel intensities of spot i on chip j. When these differences di,j are represented as colors over the area of the chip, then often the high and low ratio values are clearly concentrated in some sub-regions. An example is shown in Figure 1B. More examples are in the Supplementary Material. Because the probes for most co-regulated sets of genes are distributed widely throughout the chip, we don't expect that a biological process would generate such a pattern. Hence, such regional inhomogeneity of ratios must be a technical artifact.

### Affymetrix arrays

**Figure 2:**
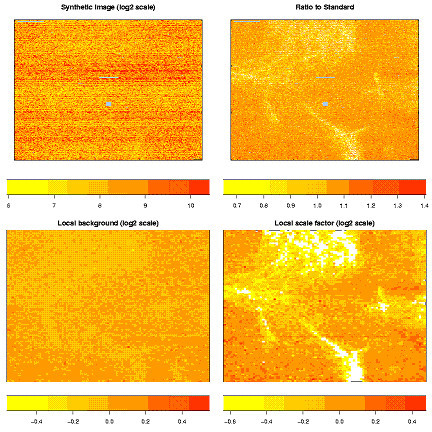
**Regional biases on an Affymetrix chip. **An Affymetrix chip is represented in log_2 _scale at upper left and the ratio of the same Affy chip to the standard chip at upper right. Each pixel of the original image represents one probe. The color legend is shown at bottom; bright red represents off-scale high, and white represents off-scale low. Blue rectangles in the upper plots indicate non-coding probes. In the lower row are images for background and scale factor for the same chip. The left plot represents the local background – the lowest levels achieved by probes on this chip relative to the lowest levels achieved by probes in the same region in other chips. The lower right plot represents the effective sensitivity or local scale factor – the log ratio of values of typically bright probes on this chip, to their values across the other chips. The scale factor captures all the variation seen in the top right image while the background image shows almost no variation. This clear separation between background artifacts and scale factor artifacts is typical in Affy chips. This chip is within the range of acceptable by our QC protocol (see discussion).

Affymetrix raw data are considerably denser (per unit area) than spotted array data, so a deeper investigation is possible. We present several types of plots here showing different aspects of bias. The first (Figure 2, upper left) shows how the raw intensity data look if we present brightness on a logarithmic scale. Ref [5] shows a similar plot. Because of the log transformation, this plot brings out detail in the low range (intensity values typically between 50 and 150). Typically, this low range contains more than half the probes on a chip. Such a plot often shows striations because probes of similar sequence are placed in rows.

Even more than with spotted array ratios, it is difficult to see subtle spatial patterns on an Affymetrix chip image because neighboring probes show such a wide range of different intensities next to one another. To make biases visible we would ideally like to compare individual slides with a standard that represents a good, uniform hybridization. Ideally, we would like to have many replicate slides of at least one representative sample and to use their average as a standard. In practice we rarely have such replicates. Hence, in the approach to be presented here, we construct a reference for the Affymetrix chips (hereafter called the 'standard' chip) by taking a trimmed mean of each probe across all chips (from the same tissue) in the experimental series. In the calculations here, we use a 20% trim, which seems to work satisfactorily. This standard chip ideally represents the probe intensities for a 'typical' sample in the experimental series – a virtual sample of the same tissue type with expression values intermediate among those of all samples in the experimental series. We then plot the differences between log values on each chip and the standard chip:

(2) d_i,j _= log2(Int_i,j_) – trim(log2(Int_i,k_)),

where i indexes the probe, j indexes the chip, and k indexes all chips; Int_i,j _is the intensity of probe i on chip j. A plot of d_ij _is shown in Figure 2 at upper right. We note that the differences {di,j} reflect discrepancies from an average, and cannot detect regions on a slide which consistently show the same bias.

Using the greater density of probes on an Affymetrix chip we can investigate in more detail how the log differences in equation (2) (i.e., the log ratios of intensities) between the sample chip and standard chip) differ from one region to another. To estimate the log difference in local background of a region, we adopt a heuristic procedure, first selecting those probes with intensities in the lowest one-fifth of probe intensities for the chip as a whole. Then we compute the 20%-trimmed mean of differences between the log_2_-intensities of the selected probes on the chip and the corresponding probes on the standard:

(3) P_j _= trim(log2(Int_i,k_)

(4) bg = trim(log2(Int_i,j_) -P_j_) | P_j _< q_P,0.2_).

Here, trim(x|S) represents the 20%-trimmed mean of the variable x restricted to the set S, and Int_i,j _represents the intensity of probe j on chip i, P_j _represents the log2-intensity of probe j on the standard, and q_P,a _represents the a-th quantile of probe intensities on the standard chip.

To compute the log_2 _scale factor, S, we use the 20%-trimmed mean among the highest 20% of probe intensities in the region:

(5) S = trim(log_2_(Int_i,j_) – P_j _| P_j _> q_P,0.8_).

We then construct heat maps of the log2 background factor (bg) and log2 scale factor (S) over the chip. When these plots are placed side by side (the bottom left and right plots in Figure 2), we see regions in which the background is raised but the scale factor is unaffected, and *vice versa*. Further examples are in the Supplementary Material. The code for making such plots for Affymetrix chips is available on our website .

### Quantitation of regional bias

It is important to have some scale on which to measure the distortions introduced by spatial effects, and to have some idea how much difference these distortions make to the final estimates of gene expression. The simplest estimate is correlation between each probe intensity or ratio and the average of its four neighbors. For spotted arrays the measure of correlation is

(6) R = < r_lm_, (r_l,m-1_+r_l,m+1_+r_l-1_,_m_+r_l+1,m_)/4 >,

where l indexes rows, m indexes columns, and r_l,m _is the log2 red-green ratio at a spot indexed by l and m. In the case where all slides use a common reference, then the difference between the log2 ratio of the spot and the average signal from that spot may be used, as described earlier. The notation <x,y> refers to the Pearson correlation between variables x and y over all values of l and m in the array. For an array with no regional bias, R would equal 0; for one with regional bias, R > 0. For Affymetrix arrays we computed R using the difference between the log2 ratio of each probe and the average signal from that probe, and we used only neighbors within rows, because neighbors within a column include the corresponding mismatch probes, which should be highly correlated with the perfect match probe.

### Measuring effect of regional biases on estimates

To test how much biases affect the expression estimates, we selected several very clean-looking chips from several different studies and systematically distorted their CEL file data by multiplying regions of various sizes by factors of 1.41 and 2, corresponding to log2 changes of 0.5 and 1.0. The distortion patterns were selected to mimic patterns that we observe in real chips. We then estimated the gene abundances using the MAS5 and RMA algorithms in the *affy *package of Bioconductor. The results did not depend much on the exact shape of the region distorted, and results were comparable using different chip types (not shown).

## Results

We investigated several hundred spotted microarrays and Affymetrix chips, from over a dozen different studies, finding noticeable bias in almost all slides and in most chips. Many of the studies included some slides or chips whose regional biases were severe enough to compromise at least part of the study.

### Spotted arrays

Using our methods we find both sharply defined, high-contrast artifacts, and diffuse regional biases. The most common sort of regional bias on spotted arrays is associated with high backgrounds of one color over a region. It is usually supposed that background subtraction removes such biases. We find, however, that the standard method of subtracting the off-spot local background from each channel does not effectively correct regional biases (see Figure 1C) and sometimes introduces them (see Figure 2). The implicit model underlying background subtraction is that the amount of non-specific DNA binding to the substrate around a spot is equal to that within the spot and additive to the target-specific binding within the spot. There are several different mechanisms that cause fluorescent signal outside of the spots – such as direct binding of dye or labeled cDNA to substrate, reflection from substrate, and binding of labeled target to smeared probe – and only some of them will contribute additively to the measured signal on spots. We think that the issue of adjustment for background needs more thought than is usually given it.

### Affymetrix chips

We find three major types of spatial artifact on Affymetrix arrays:

1) Obvious, distinct artifacts with sharp boundaries; most of those defects cover less than 5% of chip area.

2) Regional shifts in non-specific signal background: in wide areas of the chip the tenth percentile may be as much as 50% higher than the corresponding quantile over the remainder of the chip

3) Regional shifts in scale factor: in wide areas, the highest values of both PM and MM appear to be up to 50% lower than corresponding values in other areas; the scale factor appears very uneven, and shows a characteristic turbulent appearance.

We suspect that artifacts of type 2) and 3) are also present on spotted arrays, but we do not detect them as readily because the density of features is lower.

By looking in such detail, we find many irregularities in even the best Affymetrix chips. Since probes for each gene are distributed across the chip, however, a modest area (5–10% of the chip area) of affected probes is not a serious problem. A robust statistical method, such as MAS5, dChip, RMA, or the PLIER method, will down-weight those values, as described later. The problems become more serious when large (more than 20%) regions of the chip are higher in intensity than other regions by a factor of 1.5 or more. If we use a linear algorithm without outlier removal, then the values for some probes may be changed more than 1.5-fold, and the few high-intensity probes may dominate estimates for genes by these methods. If one used a linear algorithm on the log-scale then the distortion over the chip should roughly average out for each probe set. However, it is difficult to predict the effect on estimates made by a robust algorithm such as MAS5 or a linear model, such as Li-Wong, RMA or PLIER, because such methods remove outliers, and these may be found preferentially in one region of the chip. Below, we investigate empirically the effects of regional biases on gene expression estimates from robust algorithms.

### Typical measured biases

On spotted arrays we find typical correlations R between raw ratios of 0.05 to 0.1 and typical correlations using log ratios relative to the average of 0.1 to 0.2. Some slides show correlations as high as 0.6 in log ratios relative to the average.

On Affymetrix chips we find, as did Workman, only slight correlation using intensities. The correlations are much stronger for ratios of individual probes to their typical values, as instantiated in the 'standard' virtual chip. A good chip will typically show correlations in ratio relative to standard between nearest neighbors of 0.1 to 0.2. We observe the highest correlations along horizontal straight lines in the most recent generation of chips. That is so because probes with similar sequence motifs are often printed on lines (Earl Hubbell, Affymetrix Inc, personal communication), and sequence similarity may predict similar responses to many variations in conditions.

### Effect of regional biases on Affymetrix estimates

Table 1 shows the effects of simulated regional bias on gene expression for one particular sample on a Human Focus array. Systematic experiments with other array types yield comparable results.

**Table 1:** Effect of deliberate regional bias distortions on MAS5 estimates

	Region	.05	.10	.25	.50
Factor					
1.4	RMA	<.001	.011	.019	.029
	MAS5	.019	.031	.047	.063
	R	.426	.549	.69	.74
2.0	RMA	.001	.003	.039	.35
	MAS5	.034	.057	.100	.155
	R	.647	.764	.864	.89

Entries for RMA and MAS5 in the table are fractions of probe set abundance estimates (i.e., gene expression estimates) changed by more than 0.5 on a log2 scale as a consequence of the bias introduced. The rows labeled R report values of the local correlation, R.

As expected, both MAS5 and RMA are fairly robust to small distortions but, as would be expected, both methods do worse as more distortion is added to the chip images. RMA is notably more robust than MAS5 to the moderate distortions commonly found in Affymetrix chips. However, RMA does worse than MAS5 when the perturbation is most serious. A little thought makes the reason clear: RMA aims to fit the majority of the intensities on each chip well; it down-weights probes that appear too high or too low relative to the majority of others in the probe set, according to the pattern on other chips. When half of the chip is raised in intensity values relative to the other half, then roughly half the probes for each gene lie in each region. RMA fits one half well and discounts the other half.

## Discussion

### Consequences for data analysis

As described above, chips with significant regional distortions can be expected to yield gene expression estimates that differ significantly from the true values. Other, less distorted chips in a group will show expression values more indicative of the biology. Several studies that have come to the first author from leading core facilities include chips with very large spatial distortions that went undetected by the (rigorous) QC at the facility. Good intuition leads the data analyst to suspect certain outliers and to include others. However, data analysts prefer to have some objective criterion to reject outliers. In our experience, most chips that are outliers relative to their experimental groups show large regional distortions.

Rather than rely on intuition to discard outliers, we can use a systematic chip QC process to put outlier detection on a firm footing. We recommend that users note the R statistic, as defined in equation 6. We find that the R statistic is a useful guide to the degree of distortion in expression measures as summarized in Table 1. Users can decide how much distortion they are willing to live with, and select slides with R statistics accordingly. Our standard practice is to run the bias detection program in batch on a new set of chips, and to discard chips with R values exceeding 0.5. For chips with R values between 0.4 and 0.5, we scrutinize the images provided by our program, and decide whether the flaw is large and concentrated (in which case a robust procedure will limit damage to only a few probe sets), or moderate and pervasive, in which case more probe sets will be notably affected.

The discovery of systematic bias leads a statistician to try to compensate. Our QC visualization method is based on ratios, and ratios to a standard are a natural choice for normalization. Smoothing is an approach to spatial variation favored by many statisticians, and several authors have proposed compensations for microarray spatial biases using smoothed fits to bias [6-8]. However, those methods have not met with unqualified success [8,9].

We are not sanguine about the prospects for normalization by smoothing. Our observation is that the biases represented in the ratio plots show abrupt transitions from one region within a slide to another and also occur in complex filigree patterns. Often regions within the same print-tip group on a spotted microarray slide show apparent regional biases as large as do regions at greater distances on a slide (see Supplementary Figures). Sometimes there is a repeating pattern of biases in all print-tip groups. Smyth [10] has suggested that such repeating patterns derive from different quality 96-well plates used for printing the arrays. He proposes a print-order normalization, but many arrays show non-repeating, non-random patterns of bias, which can't be compensated in that way.

A reasonable question is whether regional biases in Affymetrix chips can be eliminated by comparing PM with MM. In fact, we find that a plot of log (PM_ij_) – log (MM_ij_) values for a chip, relative to the same quantities for the average chip, shows much less regional bias than does a plot of log probe intensities relative to their averages. That observation suggests that, in practice, the MAS5.0 PM correction reduces regional biases in scale factor, whereas the RMA procedure does not. In the same way, the MAS5.0 background correction reduces regional biases in background, whereas the RMA procedure does not. However our results in synthetically distorted chips suggest this advantage of the MAS5.0 procedure is telling only in the presence of strong regional bias (R>0.4).

## Conclusion

We have shown that regional biases are common on microarrays, and that in some cases they may be responsible for apparent large differences in gene expression. We have presented methods for visualizing and quantifying the levels of regional bias (and other spatial artifacts). In our judgment the most practical way to use information about regional biases on microarrays is in the quality assessment step, rather than in an attempt to compensate for it. We hope that others will use the tools we have provided at  to visualize and quantify these biases on their microarrays.

Supplementary Material is online at 

## Authors' contributions

MR conceived of the study, wrote the code, analyzed the results and drafted the manuscript. JNW suggested the perturbation study, and provided extensive and detailed comments on the manuscript. All authors read and approved the final manuscript.

**Figure 3:**
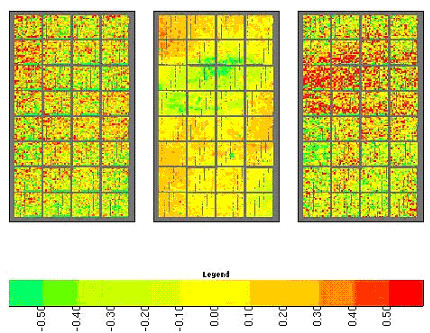
**An example where background subtraction induces regional bias. **The left image shows the raw spot ratios relative to average; the middle image shows ratios of the off-spot local background, the right image shows the ratios after background subtraction. The color legend for all three images is at bottom.
